# Prescription drug monitoring and drug overdose mortality

**DOI:** 10.1186/2197-1714-1-9

**Published:** 2014-04-24

**Authors:** Guohua Li, Joanne E Brady, Barbara H Lang, James Giglio, Hannah Wunsch, Charles DiMaggio

**Affiliations:** 1 Department of Anesthesiology, Columbia University, 622 West 168th St, PH5-505, New York, NY 10032 USA; 2 Department of Epidemiology, Columbia University, 722 West 168th St, 5th Floor, New York, NY 10032 USA; 3 Department of Emergency Medicine, Columbia University, PH1-137 New York, NY USA

**Keywords:** Policy intervention, Prescription drug, Overdose

## Abstract

**Background:**

Abuse of prescription drugs, particularly opioid analgesics, has become a major source of injury mortality and morbidity in the United States. To prevent the diversion and misuse of controlled substances, many states have implemented prescription drug monitoring programs (PDMPs). This study assessed the impact of state PDMPs on drug overdose mortality.

**Methods:**

We analyzed demographic and drug overdose mortality data for state-quarters with and without PDMPs in 50 states and the District of Columbia during 1999–2008, and estimated adjusted risk ratios (aRRs) and 95% confidence intervals (CIs) of drug overdose mortality associated with the implementation of state PDMPs through multivariable negative bionomial regression modeling.

**Results:**

During the study period, annual national death rates from drug overdose increased by 96%, from 5.7 deaths per 100,000 population in 1999 to 11.2 in 2008. The impact of PDMPs on drug overdose mortality varied greatly across states, ranging from a 35% decrease in Michigan (aRR = 0.65; 95% CI = 0.54–0.77) to a more than 3-fold increase in Nevada (aRR = 3.37; 95% CI = 2.48–4.59). Overall, implementation of PDMPs was associated with an 11% increase in drug overdose mortality (aRR = 1.11; 95% CI = 1.02–1.21).

**Conclusions:**

Implementation of PDMPs did not reduce drug overdose mortality in most states through 2008. Program enhancement that facilitates the access and use of prescription drug monitoring data systems by healthcare practitioners is needed.

**Electronic supplementary material:**

The online version of this article (doi:10.1186/2197-1714-1-9) contains supplementary material, which is available to authorized users.

## Background

Drug overdose has become a leading cause of injury mortality and morbidity in the United States, resulting in more than 34,000 deaths and 1.2 million emergency department visits each year (Centers for Disease Control and Prevention CDC 2012a; Substance Abuse and Mental Health Services Administration SAMHSA 2010a). Of the total mortality from drug overdose where a drug was specified, almost three-quarters involved one or more prescription drug (Centers for Disease Control and Prevention CDC 2011). About three-quarters of the deaths due to prescription drug overdose involved opioid analgesics (e.g., oxycodone, methadone and hydrocodone), and about one third of non-fatal emergency department visits due to prescription drug overdose involved opioid analgesics, benzodiazepines (e.g., alprazolam, clonazepam, diazepam, and lorazepam), and antidepressants (CDC 2011, SAMHSA 2010a). Collectively, opioid analgesics, benzodiazepines and antidepressants accounted for the overwhelming majority of the increase in mortality and morbidity from drug overdose in the United States in the past two decades (Centers for Disease Control and Prevention 2010; Hall et al. 2008; Paulozzi 2007).

A major contributing factor of the ongoing drug overdose epidemic is the overall increase in the availability of controlled substances. From 1991 to 2010, the annual number of prescriptions for opioid analgesics rose from approximately 75 million to almost 210 million (National Institute on Drug Abuse NIDA 2011; Volkow 2008; Volkow and McLellan 2011). Retail outpatient pharmacy sales, which in 2007 accounted for 80% of the total consumption of opioid analgesics and benzodiazepines, also showed marked increases between 1998 and 2007 (Food and Drug Administration FDA 2010). Increases in controlled substance prescribing and retail sales were associated with more use; per capita consumption of opioid analgesics quadrupled from 74 milligrams in 1997 to 369 milligrams in 2007 (Manchikanti and Singh 2008).

Per capita consumption of opioid analgesics and overdose mortality are positively correlated at the state level (Paulozzi and Ryan 2006). Medical examiner data from West Virginia indicate that of pharmaceutical overdose fatalities, 63% were attributable to drug diversion and 21% to doctor-shopping (Hall et al. 2008). The 2009 National Survey on Drug Use and Health found that 65% of nonmedical users of prescription drugs obtained these medications from friends or relatives, 20% from physicians, 5% from drug dealers or strangers, 1% from the internet, and 4% from other sources (e.g., stealing or using a fake prescription) (Substance Abuse and Mental Health Services Administration SAMHSA 2010b).

In response to the drug overdose epidemic, the federal government has intensified prevention efforts in recent years (Centers for Disease Control and Prevention CDC 2012a). One of the most important initiatives is the Harold Rogers Prescription Drug Monitoring Program (PDMP), through which the federal government has provided competitive funding since 2002 to an increasing number of states for establishing statewide electronic databases of dispensed prescriptions for controlled substances. Another important initiative, enacted in 2005, the National All Schedules Prescription Electronic Reporting Act began appropriating funds to support PDMP programs in fiscal years 2009 and 2010 (Finklea et al. 2013). The PDMP is purported to enhance the capacity of law enforcement agencies as well as public health officials to identify and investigate unusual prescribing, dispensing, and procuring patterns. Such surveillance data may also help ensure the legitimate use of controlled substances. As of April 19, 2012, 48 states and the District of Columbia have enacted PDMP legislation, including 40 states with operational PDMPs (Alliance of States with Prescription Drug Monitoring Programs 2013).

Preliminary data about the impact of state PDMPs on prescription drug consumption and overdose have been inconsistent. While some studies indicate that PDMPs were effective in reducing the consumption and abuse of opioid analgesics at the population level (Reifler et al. 2012; Simeone and Holland 2006), others found no evidence that implementing PDMPs had any appreciable impact on the overall consumption of opioids and drug overdose mortality (Paulozzi and Stier 2010; Paulozzi et al. 2011). Most of these studies were limited to data for the early period of PDMPs and few took into consideration the substantial statutory variations in state PDMPs. The present study aims to expand previous research by assessing the associations of PDMP status and PDMP characteristics with drug overdose mortality at both the national level and the state level based on data for the years 1999–2008.

## Methods

### Study design

This observational study used a “natural experiment” design, with implementation of state PDMPs as the intervention of interest. Whether and when a state implemented the intervention during the study period were not determined by random assignment or by any systematic selection process. The associations of PDMP status and PDMP characteristics with drug overdose mortality were evaluated through the analysis of state-level time series data.

### Data sources

Data on drug overdose deaths for the years 1999–2008 came from the multi-cause-of-death files of the National Center for Health Statistics. Part of the National Vital Statistics System, the multi-cause-of-death files are a census of all deaths occurring within the United States, based on death certificates compiled by individual states. Data collected from the death certificate include the decedent’s demographic characteristics, such as age, sex and race, and up to 20 causes of death coded according to the International Classification of Diseases, 10th Revision (ICD-10) (Miniño et al. 2011; National Center for Health Statistics NCHS 2011). Drug overdose deaths were identified by screening the causes of death based on ICD-10 codes X40–X44 (unintentional poisoning due to drugs) and Y10–Y14 (poisoning due to drugs of undetermined intent). Deaths of undetermined intent were incorporated in the drug overdose death counts because in some states a large portion of poisoning deaths were coded as undetermined intent (Warner and Chen 2012, Warner et al. 2013).

Annual population data for each state and the District of Columbia came from the bridged race intercensal and postcensal population estimates, developed jointly by the US Census Bureau and the National Center for Health Statistics (National Center for Health Statistics NCHS 2009). Data on annual unemployment rates for each state and the District of Columbia were obtained from the US Bureau of Labor Statistics, based on the methods used in the Current Population Survey (Bureau of Labor Statistics BLS 2008).

Information about state PDMPs was obtained from the US Drug Enforcement Administration (United States Department of Justice US DOJ 2008) and the review of PDMP characteristics conducted by the Kentucky All Schedule Prescription Electronic Reporting Program Evaluation Team (Blumenschein et al. 2010). The implementation date of a state PDMP refers to the date when electronic collection of prescription drug data began. For each implemented PDMP, four characteristics were examined: 1) type of governing agency (Department of Health, Board of Pharmacy, or Other [mostly offices related to public safety and drug control]); 2) statutory requirement for committee oversight of the program’s administration and evaluation (yes or no); 3) explicit provision that imposes no expectation on practitioners to access the statewide electronic database of dispensed prescriptions before prescribing or dispensing (yes or no); and 4) statutory authority to monitor non-controlled substances (yes or no) (Blumenschein et al. 2010).

### Statistical analysis

The unit of analysis was the state-quarter, in which quarters were delineated by the months January–March, April–June, July–September, and October–December. A state-quarter was coded as having a PDMP if the state had an operational prescription drug monitoring program any time during the quarter. During the 10-year study period, there were a total of 2040 state-quarters (10×4×51; the District of Columbia was treated as a state), including 619 in which PDMPs were operational in 31 states with varying implementation dates. The relationship between PDMP implementation and drug overdose mortality was assessed by contrasting data for state-quarters with and without PDMPs.

The adjusted risk ratios (aRRs) of drug overdose mortality associated with the implementation of PDMPs and 95% confidence intervals (CIs) were estimated through the negative binomial generalized estimating equation regression model. The negative binomial distribution accounts for greater variation and approximates the counts of drug overdose deaths within state-quarters better than the Poisson distribution (Zeleterman 2002). The generalized estimating equations were used to account for the autoregressive correlation of drug overdose deaths across time within a state (Ballinger 2004). State population counts in natural logarithms were included in the multivariable regression models as an offset term.

Possible confounding factors considered in the statistical analysis included time trend (calendar year), demographic characteristics (percent of the population that was male, percent of the population aged 35–54 years, percent of the population that was white), geographic region (Northeast, Midwest, South, and West), macroeconomic condition (unemployment rate), and accuracy of reported drug overdose deaths (type of the death investigation system, and other poisoning death rate). In this study, state death investigation systems were categorized into three groups based on the title of the medicolegal officials in the state: 1) coroners; 2) medical examiners; and 3) combination of coroners and medical examiners (Fierro 2003; Hanzlick 2007; Hanzlick and Parrish 1996). Deaths due to poisoning of other substances (“other poisoning deaths”) were identified from the multi-cause-of-death data files based on ICD-10 codes X45–X49 (unintentional poisoning by alcohol, organic solvents, pesticides, and other substances), X60–X69 (intentional self-poisoning (suicide) by drugs), and X85–X90 (assault by poisoning (homicide) by drugs).

Covariates significant at the p < 0.05 level were included in the final model. Data were analyzed using SAS 9.2 M2 (SAS Institute, Cary, NC) and Stata version 11.2 (StataCorp LP, College Station, TX).

## Results

**Figure 1:**
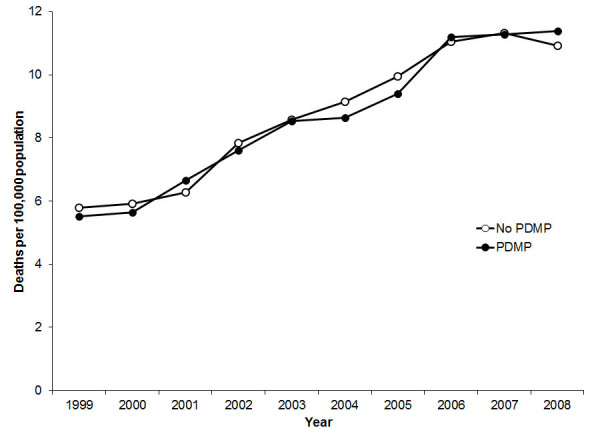
**Annual death rate from drug overdose per 100,000 population by prescription drug monitoring program implementation Status and Year, United States, 1999–2008; PDMP = Prescription drug monitoring program.**

From 1999 to 2008, annual national death rates from drug overdose per 100,000 population increased 96%, from 5.7 to 11.2. During the 10-year study period, a total of 254,507 deaths from drug overdose were recorded; 40.6% of them occurred in 619 state-quarters with operational PDMPs. The overall death rate from drug overdose in state-quarters with PDMPs was higher than in state-quarters without PDMPs (9.51 vs. 8.26 per 100,000 per year, p < .0001). The excess mortality from drug overdose was more pronounced in state-quarters with PDMPs that were governed by pharmacy boards, that had the statutory authority to monitor non-controlled substances, or that imposed no expectation on practitioners to access the statewide electronic database of dispensed prescriptions (Table 1). When the death rates from drug overdose were examined by year, there was little difference between state-quarters with and without PDMPs (Figure 1).

**Table 1:** **Number of states, number of state-quarters, number of drug overdose deaths, annualized death rate per 100,000 population, and adjusted risk ratio of drug overdose mortality by prescription drug monitoring program implementation status and characteristics, United States, 1999–2008**

PDMP Status/Characteristic	No. of states	No. of state- quarters	No. of drug overdose deaths	Death rate (95% CI)	Adjusted risk ratio ^a^(95% CI)
Without PDMP (Ref)	20	1421	151051	8.26 (8.21–8.30)	1.00
With PDMP	31	619	103456	9.51 (9.45–9.57)	1.11 (1.02–1.21)
PDMP governing agency					
Department of Health	7	163	34467	8.22 (8.13–8.30)	1.09 (0.92–1.27)
Board of Pharmacy	17	315	32198	12.10 (11.97–12.24)	1.14 (1.00–1.30)
Other	7	141	36791	9.14 (9.05–9.23)	1.05 (0.91–1.22)
Statutory requirements for committee oversight					
Yes	12	152	30366	9.97 (9.86–10.08)	1.07 (0.95–1.22)
No	19	467	73090	9.33 (9.26–9.40)	1.13 (1.02–1.26)
Explicit laws that impose no expectation on practitioners					
Yes	11	176	23403	10.50 (10.36–10.63)	1.17 (1.02–1.34)
No	20	443	80053	9.25 (9.19–9.32)	1.08 (0.96–1.20)
Statutory authority to monitor non-controlled substances					
Yes	8	89	6840	11.35 (11.09–11.63)	1.01 (0.79–1.29)
No	23	530	96616	9.40 (9.34–9.46)	1.13 (1.02–1.24)

^a^Adjusted for year, geographic region, medical examiner type, unemployment rate and poisoning mortality rate from other substances. CI = Confidence Interval; PDMP = Prescription Drug Monitoring Program.

Multivariable modeling revealed that implementation of PDMPs was associated with an 11% increase in drug overdose mortality (aRR = 1.11; 95% CI: 1.02–1.21), with the greatest increase observed in PDMPs that imposed no expectation on practitioners (aRR = 1.17; 95% CI: 1.02–1.34; Table 1). Relative to the calendar year 1999, drug overdose mortality risk increased progressively throughout the study period. Other state-level factors associated with significantly increased drug overdose mortality were being in the west region, having a medical examiner system, having an unemployment rate of 6% or higher, and having a higher poisoning mortality rate from suicides and homicides involving drugs and poisonings from substances other than drugs (Table 2). Results from the multivariable models, including the estimated effect of PDMPs on drug overdose mortality, did not change to any meaningful degree when a one-year lag was introduced to the implementation date or when the implementation date was replaced with the date when the state PDMP legislation was enacted. Results also remained consistent when opioid overdose mortality was used as the outcome or when the analysis was restricted to states with medical examiner systems.

**Table 2:** **Unadjusted and adjusted risk ratio and 95% confidence interval of drug overdose mortality according to prescription drug monitoring program implementation status and other variables, United States, 1999–2008**

Variable	Unadjusted risk ratio (95% CI)	Adjusted risk ratio (95% CI)
PDMP implemented		
No (Ref)	1.00	1.00
Yes	1.17 (1.07–1.27)	1.11 (1.02–1.21)
Year		
1999 (Ref)	1.00	1.00
2000	1.08 (1.01–1.15)	1.08 (1.02–1.15)
2001	1.17 (1.04–1.32)	1.13 (1.02–1.24)
2002	1.38 (1.23–1.55)	1.25 (1.02–1.38)
2003	1.57 (1.38–1.79)	1.38 (1.24–1.55)
2004	1.70 (1.50–1.93)	1.54 (1.39–1.70)
2005	1.84 (1.62–2.08)	1.67 (1.50–1.86)
2006	1.99 (1.72–2.30)	1.87 (1.66–2.10)
2007	2.06 (1.81–2.34)	1.81 (1.60–2.04)
2008	2.20 (1.93–2.50)	1.71 (1.51–1.92)
Region		
East (Ref)	1.00	1.00
Midwest	0.70 (0.55–0.89)	0.77 (0.59–1.01)
South	1.07 (0.89–1.27)	1.07 (0.89–1.29)
West	1.22 (0.99–1.51)	1.20 (0.98–1.46)
Death investigation system type^a^		
Coroner (Ref)	1.00	1.00
Coroner/Medical examiner	1.13 (0.75–1.71)	1.05 (0.77–1.42)
Medical examiner	1.38 (0.91–2.10)	1.28 (0.93–1.77)
Unemployment rate		
<6% (Ref)	1.00	1.00
≥6%	1.05 (0.99–1.23)	1.07 (1.03–1.11)
Suicides and homicides involving drugs and poisoning from substances other than drugs	1.19 (1.13–1.26)	1.16 (1.10–1.23)

^a^States with a coroner system were ID, IN, KS, LA, ND, NE, NV, SD, WY; States with a combination of coroner and medical examiner systems were AK, AL, AR, CA, CO, GA, HI, IL, KY, MN, MO, MS, MT, NY, OH, PA, SC, TX, WA, WI; and states with a medical examiner system were AZ, CT, DC, DE, FL, IA, MA, MD, ME, MI, NC, NH, NJ, NM, OK, OR, RI, TN, UT, VT, VA, WV (Hanzlick 2007; Hanzlick and Parrish 1996; Standing Bear 2012). CI = confidence interval; PDMP = Prescription Drug Monitoring Program; RR = risk ratio.

The impact of PDMPs on drug overdose mortality varied markedly across states (Table 3). Implementation of PDMPs was associated with a significantly decreased risk of drug overdose mortality in three states, no significant effect in 11 states, and a significantly increased risk in 17 states (Table 3). The greatest reduction in drug overdose mortality associated with PDMP implementation was in Michigan (aRR = 0.65; 95% CI: 0.54–0.77) and the largest increase in drug overdose mortality associated with PDMP implementation was in Nevada (aRR = 3.37; 95% CI: 2.48–4.59).

**Table 3:** **Adjusted risk ratio and 95% confidence interval of drug overdose mortality associated with the implementation of the prescription drug monitoring program by State, United States, 1999–2008**

State	Adjusted risk ratio ^a^(95% CI)	State	Adjusted risk ratio ^a^(95% CI)
Michigan	0.65 (0.54–0.77)	Ohio	1.15 (1.03–1.29)
Virginia	0.77 (0.66–0.89)	Alabama	1.16 (1.02–1.33)
New York	0.86 (0.74–0.99)	Louisiana	1.18 (0.84–1.65)
Maine	0.91 (0.80–1.04)	Oklahoma	1.18 (1.03–1.36)
Mississippi	0.95 (0.84–1.09)	Hawaii	1.28 (1.07–1.52)
North Carolina	0.95 (0.86–1.06)	Idaho	1.35 (1.03–1.77)
California	0.96 (0.85–1.09)	New Mexico	1.35 (1.18–1.56)
Massachusetts	0.96 (0.82–1.12)	Kentucky	1.42 (1.24–1.64)
Texas	1.00 (0.87–1.16)	Indiana	1.44 (1.20–1.73)
Arizona	1.02 (0.94–1.10)	Utah	1.47 (1.16–1.87)
South Carolina	1.04 (0.93–1.16)	Pennsylvania	1.49 (1.33–1.68)
Tennessee	1.05 (0.94–1.17)	West Virginia	1.49 (1.28–1.73)
Illinois	1.11 (0.98–1.25)	Wyoming	1.53 (1.23–1.89)
Rhode Island	1.11 (0.91–1.35)	North Dakota	1.72 (1.43–2.07)
Colorado	1.12 (1.00–1.25)	Nevada	3.37 (2.48–4.59)
Connecticut	1.12 (1.00–1.25)		

^a^Adjusted for year, geographic region, medical examiner type, unemployment rate and poisoning mortality rate from other substances; and estimated separately for each state using state-quarter data for the individual state and those without an operational Prescription Drug Monitoring Program throughout the study period (AK, AR, DC, DE, FL, GA, IA, KS, MD, MN, MT, MO, NE, NH, NJ, OR, SD, VT, WA, and WI).

CI = confidence interval.

## Discussion

Results of this study indicate that implementing PDMPs did not reduce drug overdose mortality in most states through 2008. This finding is consistent with previous reports (Paulozzi et al. 2011; Brady et al. 2014). Paulozzi et al. (2011) analyzed annual state-level mortality and drug consumption data from 1999 to 2005 and found no discernible impact of PDMPs on either drug overdose mortality or per capita consumption of opioid analgesics.

The lack of effectiveness of state PDMPs in reducing drug overdose mortality could be attributed to several factors. Foremost is the severely limited use of the electronic databases of dispensed prescriptions by physicians and pharmacists due to difficult accessibility and insufficient incentives (Green et al. 2011). Use of state PDMP databases by healthcare professionals is further hampered by liability concerns. Of the 31 implemented PDMPs included in this study, 11 contained provisions exempting practitioners from the obligation to access the state PDMP database, and these 11 PDMPs appeared to be associated with greater drug overdose mortality than other PDMPs. Other factors limiting the effectiveness of PDMPs include barriers to interstate data sharing and inadequate healthcare provider training on prescribing controlled substances (Manchikanti 2007; McLellan and Turner 2008; Volkow and McLellan 2011).

It is possible that the greater drug overdose mortality associated with PDMPs was due to residual confounding from factors that were controlled for and imperfectly measured, and from unmeasured variables. For example, controlling for census regions may not adequately control for geographic variations in overdose death between states in different geographic regions. Specifically, the cultural region of Appalachia has been greatly affected by the overdose epidemic and spans 13 states and 3 census regions. Thus, controlling for census region may not adequately control for the drug overdose epidemic in Appalachia. However, it is also plausible that the increased risk of drug overdose mortality might reflect unintended criminal justice consequences of the intervention. PDMPs were designed and implemented primarily for law enforcement purposes. During the study period, state PDMP databases served mostly as a tool for drug control agencies to identify and investigate healthcare practitioners and patients engaging in fraudulent activities. It is evident that law enforcement alone is insufficient to control drug abuse and might in fact be counterproductive because drug users could be forced to riskier practices (Cooper et al. 2005) and are especially susceptible to fatal overdose during the two weeks immediately after release from prison (Binswanger et al. 2007; Lim et al. 2012).

While state PDMPs share substantial uniformity in the structure of centralized statewide data systems and basic elements of electronically transmitted prescription data, they differ in many key features, such as statutory requirements for committee oversight, and for data access and reporting. This study examined four of these features— type of governing agency, requirement for committee oversight, explicit provision exempting practitioners from the obligation to access the state PDMP database, and statutory authority to monitor non-controlled substances—and found that none of them had a significant protective effect on drug overdose mortality. Some of these features, such as an explicit provision exempting healthcare practitioners from the obligation to use the state PDMP database, appear to be detrimental. A recent study that examined the comparative effectiveness of “proactive” PDMPs (i.e., those providing unsolicited reports to healthcare practitioners and law enforcement agencies) and other PDMPs did not find any significant difference in drug overdose mortality between the two groups (Paulozzi et al. 2011). These findings suggest that inadequate utilization of the PDMP database by healthcare practitioners may largely void the potential health benefit of the program, rendering program characteristics essentially irrelevant.

Although variations in state PDMPs have been well documented (Blumenschein et al. 2010), there is limited information about the effectiveness of PDMPs for individual states (Office of National Drug Control Policy ONDCP 2011; Prescription Drug Monitoring Program Center of Excellence at Brandeis 2013). There are now best practice recommendations that have been developed to help states analyze PDMP data (Clark et al. 2012). The state-level analysis in this study indicates that PDMPs implemented in Michigan, Virginia, and New York are most effective and those implemented in Nevada, North Dakota, Wyoming, West Virginia, and Pennsylvania are least effective. These findings may facilitate further research through case studies and other qualitative methods to delineate program elements and implementation processes responsible for the divergent effectiveness of the state PDMPs.

The above findings should be interpreted with caution for several reasons. First, results from this observational study are susceptible to information bias and unmeasured confounding. Although the multi-cause-of-death data files represent a census of all deaths occurring within the United States, the accuracy in the recorded drug overdose deaths and including those involving opioids may vary from state to state (Warner and Chen 2012; Warner et al. 2013). Opioid analgesic rates are subject to differential classification of opioids (Warner et al. 2013). There is conflicting evidence regarding the accuracy of the coding and determination of drug overdose deaths (Landen et al. 2003, Manini et al. 2011). This study confirms that reported drug overdose mortality is significantly higher in states where injury deaths are investigated by medical examiners than in states where such deaths are investigated by coroners. Whereas adjusting for the type of the death investigation system is necessary, it is likely insufficient to control for information bias in the drug overdose mortality data. Second, this study is limited to data for the years 1999–2008 and the findings should not be extrapolated beyond 2008 given the extensive changes in state PDMPs in recent years. Since 2009, 13 states have received supplemental funding through the National All Schedules Prescription Electronic Reporting Act to bolster their PDMPs, such as the accessibility of prescription data and the capacity for interstate information exchange (Alliance of States with Prescription Drug Monitoring Programs 2013). Third, drug overdose mortality in this study is based on ICD-10 codes X40–X44 and Y10–Y14, which include overdose deaths involving prescription drugs as well as overdose deaths involving other substances. While prescription drugs are not always identified in death certificate data and 60% of overdose deaths are thought to be related to prescription drugs, drug overdose mortality in this study may include deaths caused by methamphetamine, cocaine, heroin, and other illicit drugs, which are beyond the purview of PDMPs. There is preliminary evidence that as prescription drugs become more difficult to procure, illicit drugs such as heroin may be substituted. Because the outcome definition in this analysis does not distinguish between illicit and prescription drugs, if there were fewer deaths resulting from prescription drugs with the implementation of PDMP programs, this effect would not be detected in this analysis. Fourth, this study controlled for ICD-10 codes X60–X64 (suicide by “drug” overdose) and X86 (homicide by “drug” overdose), as well as overdose/poisoning of other substances (e.g., X65–X69), which may or may not involve drugs. Suicide by drug overdose and homicide by drug overdose are likely more relevant to PDMPs than poisoning from other substances. Finally, this study assessed the possible impact of implementing PDMPs on drug overdose mortality only. It is noteworthy that the primary purpose of PDMPs is to prevent diversion of controlled substances, not drug overdose per se. Although the majority of drug overdose deaths involve the use of diverted drugs (Hall et al. 2008), targeting drug diversion alone through law enforcement may not be sufficient to reduce drug overdose mortality. To control the ongoing drug overdose epidemic, a multifaceted approach is needed. Recent reports and commentaries have called for using prescription data and insurance restrictions to help prevent procurement of controlled substance prescriptions from multiple healthcare providers, augmenting healthcare provider training on pain management, implementing evidence-based guidelines for the management of pain, and improving access to drug treatment and expanding community-based harm reduction programs (Centers for Disease Control and Prevention 2012b; United Nations Office on Drugs and Crime UN ODC 2013; Volkow and McLellan 2011).

## Conclusions

Despite its limitations, this study adds compelling evidence that up to 2008, implementing PDMPs had not reduced drug overdose mortality in most states. The lack of effectiveness is likely due to the severely limited utilization of state PDMP data systems by healthcare practitioners. Enhancing the capacity and accessibility of state PDMPs is imperative to facilitate integrating the prescription drug monitoring data system into clinical practice. Continuing efforts to refine and strengthen prevention programs are necessary to effectively reduce drug overdose mortality and morbidity.

## Authors’ original submitted files for images

Below are the links to the authors’ original submitted files for images. Authors’ original file for figure 1

## References

[CR1] Alliance of States with Prescription Drug Monitoring Programs (2013). Alliance of States with Prescription Drug Monitoring Programs.

[CR2] Ballinger G (2004). Using generalized estimating equations for longitudinal data analysis. Organ Res Methods.

[CR3] Binswanger IA, Stern MF, Deyo RA, Heagerty PJ, Cheadle A, Elmore JG, Koepsell TD (2007). Release from prison — a high risk of death for former inmates. N Engl J Med.

[CR4] Blumenschein K, Fink JL, Freeman PR, James K, Kirsch KL, Steinke DT, Talbert J (2010). Kentucky All Schedule Prescription Electronic Reporting Program (KASPER) Evaluation Team. Review of Prescription Drug Monitoring Programs in the United States.

[CR5] Brady JE, Wunsch H, DiMaggio CJ, Lang BH, Giglio J, Li G (2014). Prescription drug monitoring and dispensing of prescription opioids. Public Health Rep.

[CR6] Bureau of Labor Statistics (BLS) (2008). Local Area Unemployment Statistics.

[CR7] Centers for Disease Control and Prevention (CDC) (2012a). CDC grand rounds: prescription drug overdoses - a U.S. epidemic. MMWR Morb Mortal Wkly Rep.

[CR8] Centers for Disease Control and Prevention (CDC) (2011). Vital signs: overdoses of prescription opioid pain relievers---United States, 1999--2008. MMWR Morb Mortal Wkly Rep.

[CR9] Centers for Disease Control and Prevention (2012b). Community-based opioid overdose prevention programs providing naloxone – United States, 2010. MMWR Morb Mortal Wkly Rep.

[CR10] Centers for Disease Control and Prevention (2010). Emergency department visits involving nonmedical use of selected prescription drugs–United States, 2004–2008. MMWR Morb Mortal Wkly Rep.

[CR11] Clark T, Eadie J, Knue P, Kreiner P, Strickler G (2012). Prescription Drug Monitoring Programs: An Assessment of the Evidence for Best Practices. The Prescription Drug Monitoring Program Center of Excellence.

[CR12] Cooper H, Moore L, Gruskin S, Krieger N (2005). The impact of a police drug crackdown on drug injectors’ ability to practice harm reduction: a qualitative study. Soc Sci Med.

[CR13] Fierro M (2003). Comparing Medical Examiner and Coroner Systems: advantages and Disadvantages of the Medical Examiner System. Medicolegal Death Investigation System: Workshop Summary.

[CR14] Finklea KM, Bagalman E, Sacco LN (2013). Prescription Drug Monitoring Programs. Congressional Research Service.

[CR15] Food and Drug Administration (FDA) (2010). Risk Evaluation and Mitigation Strategies (REMS) for Extended-Release and Long Acting Analgesics. Talk Presented at: The Joint Meeting of the Anesthetic and Life Support Drugs Advisory Committee and the Drug Safety and Risk Management Advisory Committee.

[CR16] Green TC, Zaller N, Rich J, Bowman S, Friedmann P (2011). Revisiting Paulozzi et al.'s "Prescription drug monitoring programs and death rates from drug overdose". Pain Med.

[CR17] Hall AJ, Logan JE, Toblin RL, Kaplan JA, Kraner JC, Bixler D, Crosby AE, Paulozzi LJ (2008). Patterns of abuse among unintentional pharmaceutical overdose fatalities. JAMA.

[CR18] Hanzlick R, Parrish RG (1996). The role of medical examiners and coroners in public health surveillance and epidemiologic research. Annu Rev Public Health.

[CR19] Hanzlick R (2007). The conversion of coroner systems to medical examiner systems in the United States: a lull in the action. Am J Forensic Med Pathol.

[CR20] Landen MG, Castle S, Nolte KB, Gonzales M, Escobedo LG, Chatterjee BF, Johnson K, Sewell CM (2003). Methodological issues in the surveillance of poisoning, illicit drug overdose, and heroin overdose deaths in New Mexico. Am J Epidemiol.

[CR21] Lim S, Seligson AL, Parvez FM, Luther CW, Mavinkurve MP, Binswanger IA, Kerker BD (2012). Risks of drug-related death, suicide, and homicide during the immediate post-release period among people released from New York City jails, 2001–2005. Am J Epidemiol.

[CR22] Manchikanti L, Singh A (2008). Therapeutic opioids: a ten-year perspective on the complexities and complications of the escalating use, abuse, and nonmedical use of opioids. Pain Physician.

[CR23] Manchikanti L (2007). National drug control policy and prescription drug abuse: facts and fallacies. Pain Physician.

[CR24] Manini AF, Nelson LS, Olsen D, Vlahov D, Hoffman RS (2011). Medical examiner and medical toxicologist agreement on cause of death. Forensic Sci Int.

[CR25] McLellan AT, Turner B (2008). Prescription opioids, overdose deaths, and physician responsibility. JAMA.

[CR26] Miniño AM, Murphy SL, Xu JQ, Kochanek KD (2011). Deaths: Final data for 2008. National vital statistics reports; vol 59 no 10.

[CR27] National Center for Health Statistics (NCHS) (2011). 2008 Documentation Initial Release: Mortality Multiple Cause-of-Death Public Use Record.

[CR28] National Center for Health Statistics (NCHS) (2009). CDC Wonder [database online].

[CR29] National Institute on Drug Abuse (NIDA) (2011). Topics in Brief: Prescription Drug Abuse.

[CR30] Office of National Drug Control Policy (ONDCP) (2011). Prescription Drug Monitoring Programs. Office of National Drug Control Policy.

[CR31] Paulozzi LJ, Kilbourne EM, Desai HA (2011). Prescription drug monitoring programs and death rates from drug overdose. Pain Med.

[CR32] Paulozzi LJ, Ryan GW (2006). Opioid analgesics and rates of fatal drug poisoning in the United States. Am J Prev Med.

[CR33] Paulozzi LJ, Stier LD (2010). Prescription drug laws, drug overdoses, and drug sales in New York and Pennsylvania. J Public Health Policy.

[CR34] Paulozzi LJ (2007). The Epidemiology of Unintentional Drug Poisoning in the United States. Talk Presented at: State Epidemiology Outcomes Workgroup Audio Conference.

[CR35] Prescription Drug Monitoring Program Center of Excellence at Brandeis (2013). Briefing on PDMP Effectiveness. Prescription Drug Monitoring Program Center of Excellence at Brandeis.

[CR36] Reifler LM, Droz D, Bailey JE, Schnoll SH, Fant R, Dart RC, Bucher Bartelson B (2012). Do prescription monitoring programs impact state trends in opioid abuse/misuse?. Pain Med.

[CR37] Simeone R, Holland L (2006). An Evaluation of Prescription Drug Monitoring Programs.

[CR38] Standing Bear ZG (2012). Conflicts of Interest in US Coroner Systems.

[CR39] Substance Abuse and Mental Health Services Administration (SAMHSA) (2010a). Center for Behavioral Health Statistics and Quality (formerly the Office of Applied Studies). The DAWN Report: Highlights of the 2009 Drug Abuse Warning Network (DAWN) Findings on Drug-Related Emergency Department Visits.

[CR40] Substance Abuse and Mental Health Services Administration (SAMHSA) (2010b). Center for Behavioral Health Statistics and Quality (formerly the Office of Applied Studies). RTI, Research Triangle Park. Results from the 2009 National Survey on Drug Use and Health: National Findings.

[CR41] United Nations Office on Drugs and Crime (UN ODC) (2013). Opioid overdose: preventing and reducing opioid overdose mortality. United Nations.

[CR42] United States Department of Justice (US DOJ) (2008). State Prescription Drug Monitoring Programs.

[CR43] Volkow ND, McLellan TA (2011). Curtailing diversion and abuse of opioid analgesics without jeopardizing pain treatment. JAMA.

[CR44] Volkow ND (2008). Scientific Research on Prescription Drug Abuse: Statement Before the Subcommittee on Crime and Drugs of the Senate Judiciary Committee, 110th Cong., 2nd Sess.

[CR45] Warner M, Chen L, Li G, Baker SP (2012). Surveillance of injury mortality. Injury Research: Theories, Methods, and Approaches.

[CR46] Warner M, Paulozzi LJ, Nolte K, Davis GG, Nelson LS (2013). State variation in certifying manner of death and drugs involved in drug intoxication deaths. Acad Forensic Pathol.

[CR47] Zeleterman D (2002). Advanced Log-Linear Models Using SAS.

