# Supplementary figures and images for: Prescription drug monitoring and drug overdose mortality
Source: Inj Epidemiol. 2014 Apr 24;1(1):9. doi: 10.1186/2197-1714-1-9 (PMC5005551; doi:10.1186/2197-1714-1-9)

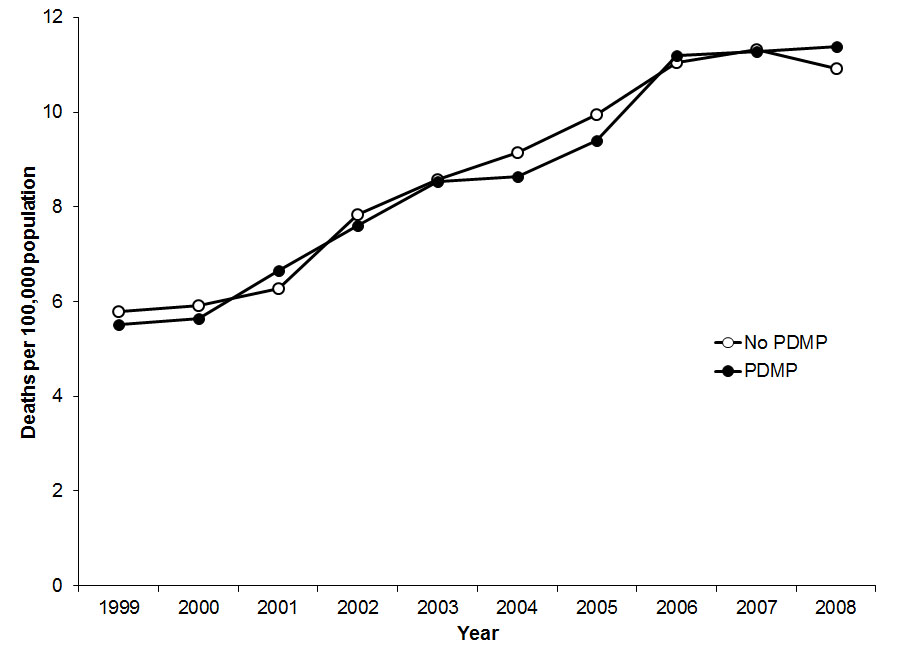

Supplement: Supplementary file 1 — Authors’ original file for figure 1 [file 40621_2013_9_MOESM1_ESM.jpeg]
